# Mastopathic-Type Breast Fibroadenomas Can Have Indistinct Margins on Ultrasound: A Case Report

**DOI:** 10.7759/cureus.82393

**Published:** 2025-04-16

**Authors:** Nozomi Uozumi, Shoji Oura

**Affiliations:** 1 Department of Surgery, Kishiwada Tokushukai Hospital, Kishiwada, JPN

**Keywords:** breast mass, fibrocystic disease, indistinct margins, mastopathic-type fibroadenoma, ultrasound

## Abstract

Fibroadenoma is one of the most common benign breast tumors and exclusively has clear margins regardless of subtypes. We herein report a mastopathic-type fibroadenoma with indistinct margins on ultrasound. A 53-year-old postmenopausal woman with cecum cancer presented with a right breast mass on staging computed tomography (CT) for the cecum cancer. Mammography only suggested the presence of a breast mass and could not contribute to image diagnosis due to the dense breast. Ultrasound showed that the mass had indistinct margins, multiple dotted or linear hyperechoic foci against the background low echoes, and posterior acoustic enhancement. Magnetic resonance imaging (MRI) depicted that the mass had distinct margins and showed low signals on T1-weighted images, high signals on T2-weighted images, and a persistent pattern on subtraction MRI. Core needle biopsy pathologically showed hyperplasia of the ductal epithelium without any malignant findings. The patient, therefore, underwent a lumpectomy of the breast mass during the cecum cancer operation. Postoperative pathological study showed that the oval mass had clear but ruffled tumor borders, many fat cell clusters near the mass edges, stromal cell proliferation, and plentiful glands showing a blunt duct adenosis-like pattern with focal papillomatosis. These pathological findings led us to the diagnosis of mastopathic-type fibroadenoma. Diagnostic physicians should note that mastopathic-type fibroadenomas can have various pathological components of fibrocystic disease and therefore can have indistinct margins on ultrasound.

## Introduction

Breast cancers overwhelmingly present irregular-shaped masses with indistinct margins. Hence, spiculated, irregular, high-density masses on mammography are judged as Breast Imaging Reporting and Data System (BI-RADS) category 5, i.e., highly suggestive of malignancy. However, some breast cancers, naturally not frequently, can be depicted as circumscribed masses [1]. Conversely, benign breast tumors generally show round or oval masses with circumscribed margins [2,3]. In other words, it is extremely rare for benign tumors to have indistinct margins. Clear tumor margins, therefore, are regarded as necessary but not sufficient conditions for the diagnosis of benign tumors.

Breast fibroadenomas generally present as well-defined, mobile masses, account for one-half of all breast biopsies, and are benign tumors that occur more frequently in younger women than in those with breast cancer [4]. Younger women are more likely to have dense breasts than older women, often making image diagnosis of breast tumors more difficult. It, therefore, is very important for diagnostic physicians to become familiar with fibroadenoma images in daily clinical practice.

It is well known that estrogen is strongly associated with the growth of all breast fibroadenomas, and mutation of *MED12*, essential for activating CDK8 kinase and also playing an important role in the development of uterine leiomyomas, is found in many of them [5]. Breast fibroadenomas are clinically classified into four subtypes: intracanalicular, pericanalicular, organoid, and mastopathic [5]. The first and second subtypes are very familiar to clinicians. The third subtype is known to have epithelial components showing lobular structures of the mammary gland. The last subtype generally has epithelial components demonstrating fibrocystic disease-like structures and therefore is also well known to be frequently overdiagnosed on cytological evaluation. Each subtype is well known to have different characteristic pathological findings. They, however, all generally have a common characteristic to show clear tumor margins with various images.

We herein report a case of mastopathic-type fibroadenoma that showed indistinct margins on ultrasound.

## Case presentation

**Figure 1:**
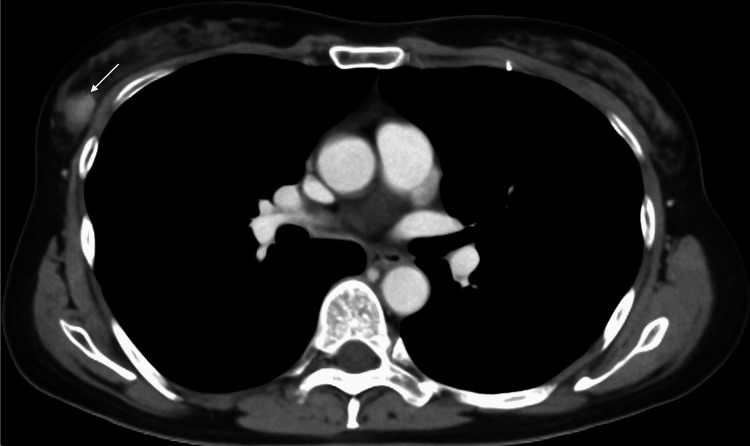
CT findings CT showed a well-enhanced oval mass with circumscribed margins in the right mammary gland (arrow). CT: computed tomography

A 53-year-old postmenopausal woman with a positive fecal occult blood test underwent colonoscopy, leading to the diagnosis of cecum cancer. The patient, therefore, was referred to our hospital for surgical treatment. Staging computed tomography (CT) incidentally revealed a non-palpable oval mass with circumscribed margins in her right breast (Figure 1).

**Figure 2:**
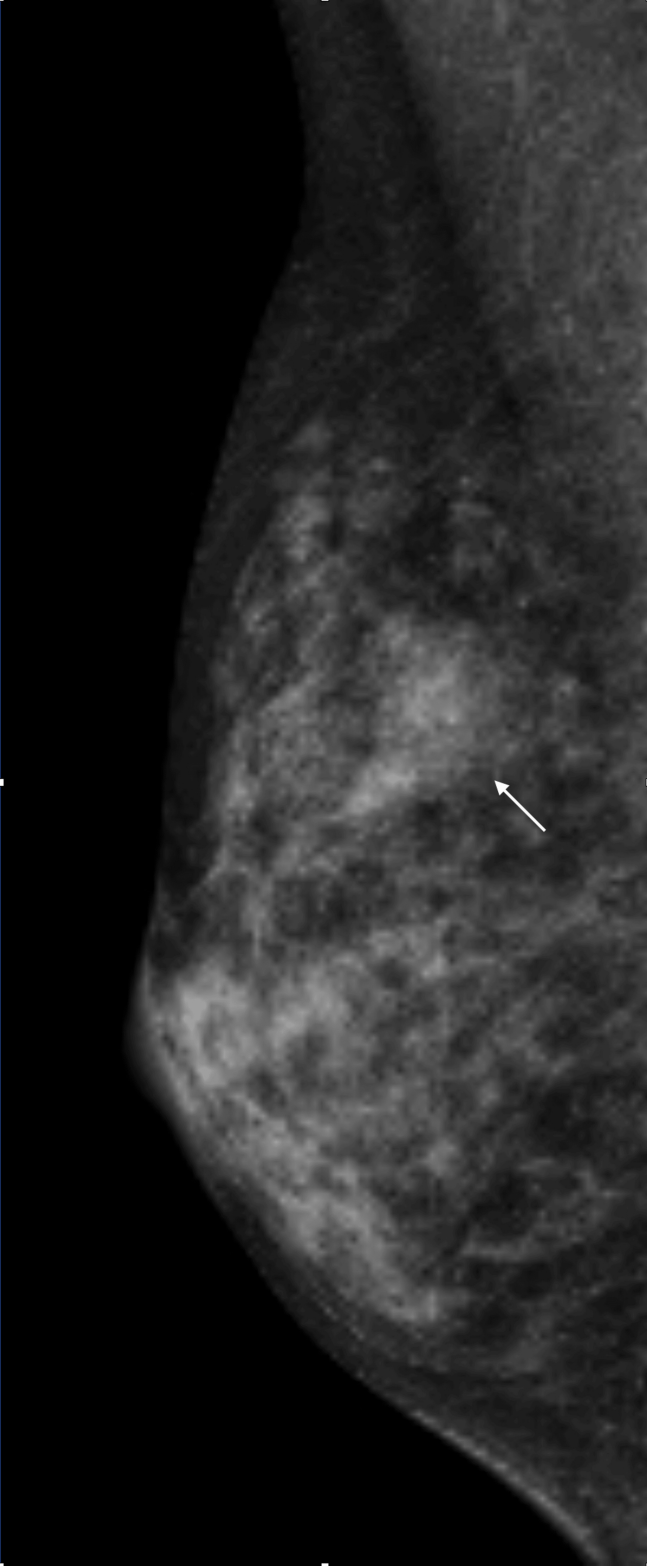
Mammography findings Dense breast obscured the margins of the right breast mass (arrow) on the mediolateral oblique mammogram.

Mammography showed background dense breast and a mass image in the upper outer quadrant of her right breast but could not contribute to determine whether the mass was benign or malignant (Figure 2).

**Figure 3:**
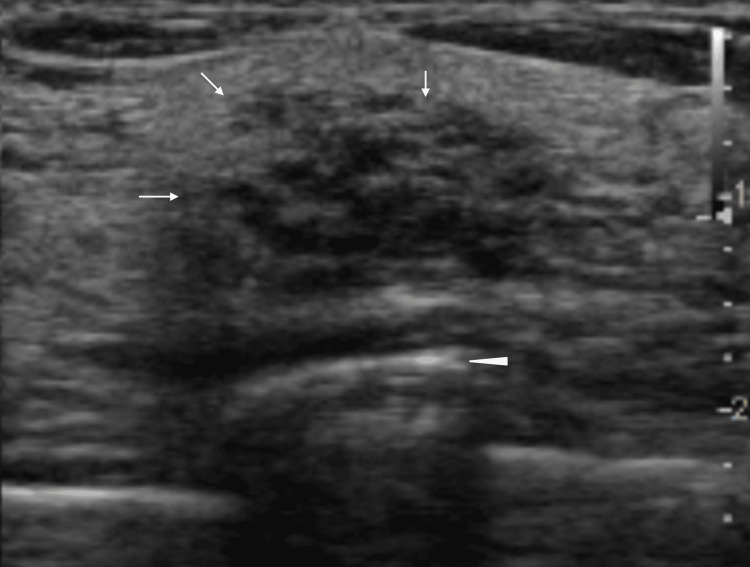
Ultrasound findings Ultrasound showed that the mass had indistinct margins (arrows), hyperechoic dots/lines, and slight posterior acoustic enhancement (arrowhead).

Ultrasound showed that the mass had indistinct margins, multiple dotted or linear hyperechoic foci against the background low echoes, and posterior acoustic enhancement (Figure 3).

**Figure 4:**
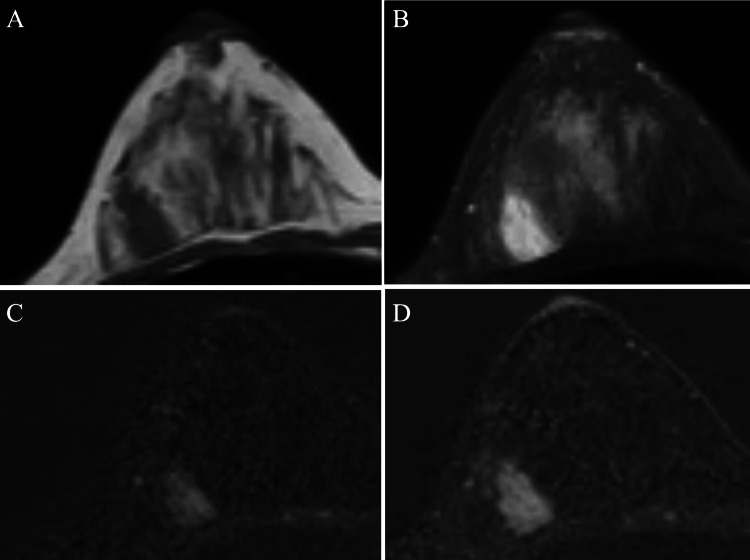
MRI findings MRI showed a well-circumscribed mass on T1-weighted (A), T2-weighted (B), and subtraction (C and D) images. In addition, subtraction images showed a homogenous and persistent enhancement pattern of the tumor (C and D). MRI: magnetic resonance imaging

Magnetic resonance imaging (MRI) showed that the mass had distinct margins and showed low signals on T1-weighted images, high signals on T2-weighted images, and a persistent enhancement pattern on subtraction MRI (Figure 4).

**Figure 5:**
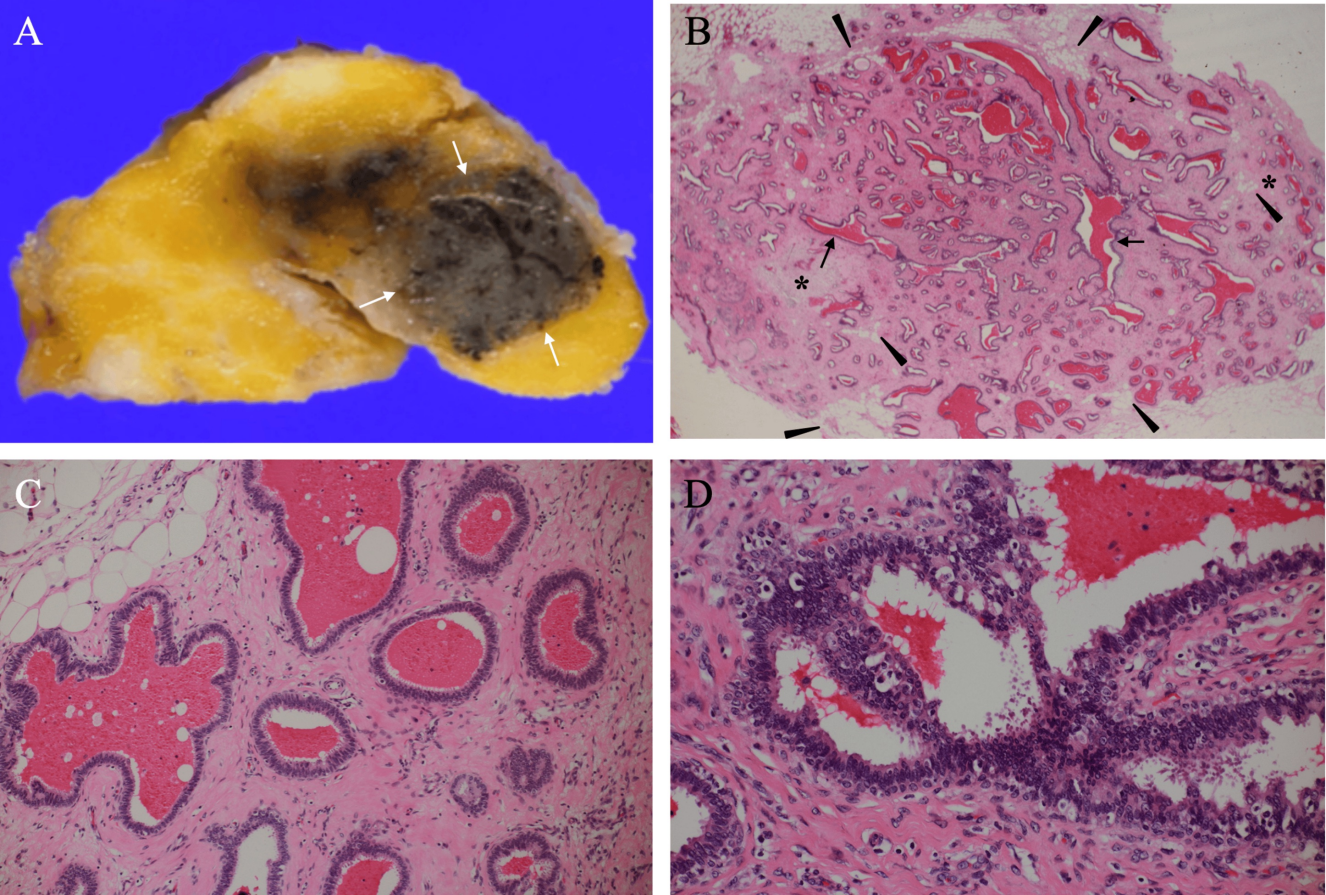
Pathological findings (A) The bisected mass showed that it was dark brownish and had clear margins (arrows). (B) Magnified view showed that the tumor had stromal cell proliferation (asterisks), no long smooth margins, entanglement of many fat cells near the mass borders (arrowheads), and plentiful tubule-forming structures in the mass with (arrows) or without hemorrhage presumably due to core needle biopsy. (C) Magnified view showed multiple clusters of small, slightly dilated acini-like ducts, i.e., typical of blunt duct adenosis. (D) Magnified view showed focal ductal proliferation, as seen in intraductal papillomatosis.

Core needle biopsy pathologically showed only hyperplasia of the ductal epithelium without any malignant findings. The patient, therefore, underwent lumpectomy for the breast mass at the cecum cancer operation to get both a definitive diagnosis and cure of the presumed benign tumor. The bisected cut surface of the mass had clear borders and was dark brownish, probably due to the core needle biopsy-induced intra-tumoral bleeding. Postoperative pathological study showed that the mass was oval and had clear but ruffled borders, i.e., no long smooth margins. In addition, the tumor had many fat cell clusters near the mass edges, stromal cell proliferation, and plentiful glands showing a blunt duct adenosis-like pattern, both with bleeding in the vast majority of them and focal papillomatosis (Figure 5).

These pathological findings led us to the diagnosis of mastopathic-type fibroadenoma. After the lumpectomy, the patient underwent an ileocecal resection with regional node dissection for the cecum cancer. The patient recovered uneventfully and was discharged on the seventh day after the operation. She began to receive postoperative adjuvant capecitabine-containing chemotherapy on an outpatient basis but stopped it very shortly due to severe chemotherapy-induced side effects, i.e., grade 3 malaise.

## Discussion

The World Health Organization defines fibroadenoma as a "circumscribed benign neoplasm of the terminal duct lobular unit with biphasic proliferation of epithelial and stromal components" [6]. In fact, the bisected cut surface of the resected mass had macroscopically clear borders. The dense breast in this case obscured the tumor margins on mammography, which is frequently experienced in clinical practice. CT and MRI, however, showed that the mass clearly had well-defined margins.

Fibroadenomas have a large amount of fibrous components, which are generally accompanied by stromal edema in premenopausal cases [7]. Stromal edema, therefore, makes the fibroadenoma, regardless of fibroadenoma subtypes, hypo-intense on T1-weighted images and hyper-intense on T2-weighted images [8]. In addition, fibrous components generate a plateau or persistent enhancement pattern on subtraction MRI [9]. This patient was postmenopausal but had just entered the postmenopausal status, therefore showing the typical MRI images similar to those of fibroadenomas in premenopausal patients.

The *MED12* mutation is observed in more than half of breast fibroadenomas [10]. Of the four main subtypes of breast fibroadenomas, the *MED12* mutation is closely correlated with intracanalicular fibroadenomas [5]. The driver gene *MED12* mutation is also associated with the growth of uterine leiomyomas [11]. This fact, together with the correlation between *MED12* mutations and intracanalicular type fibroadenomas [5], suggests that *MED12* is more closely related to the proliferation of fibrous components than that of epithelial components. Conversely, mastopathic-type fibroadenomas have less correlation with *MED12* mutations and a low tendency for the proliferation of fibrous components, possibly allowing them to have ruffled borders and fat cell clusters near the tumor margins encompassed by peri-tumoral fat. These speculations explain why mastopathic-type fibroadenoma could have had indistinct margins on ultrasound.

It is well known that the reflection of ultrasound waves forms tumor shapes, whereas their backscattering generates internal echoes of the tumor [12,13]. The reflection of ultrasound waves needs smooth boundary interfaces, which are much larger than the ultrasound wavelength. However, in addition to the ruffled tumor borders, the presence of many fat cell clusters near the tumor edges hampered the creation of clear tumor margins on ultrasound. In addition, a large number of ductal proliferations, such as duct papillomatosis or blunt duct adenosis distributing very close to the mass borders, further obscured the mass margins on ultrasound.

Diagnostic physicians, therefore, should note that some mastopathic-type fibroadenomas can have obscured mass margins on ultrasound. Breast surgeons should follow up the mass with indistinct borders rather than immediately resect it when the mass depicts typical fibroadenoma images on MRI and shows cytological/pathological findings of ductal hyperplasia.

## Conclusions

Fibroadenomas typically have clear tumor borders; however, mastopathic subtypes may present with atypical features such as indistinct margins on ultrasound, despite being benign. Although surgical excision was performed due to the synchronous abdominal surgery, similar cases may be managed conservatively if imaging and biopsy are concordantly benign.
